# Concerns Regarding Validity of the Use of Bean Extract-Based Gargle for COVID-19 Diagnosis

**DOI:** 10.1128/spectrum.00738-22

**Published:** 2022-07-05

**Authors:** Ki Ho Hong, Heungsup Sung

**Affiliations:** a Department of Laboratory Medicine, Yonsei University College of Medicine, Seoul, South Korea; b Department of Laboratory Medicine, Asan Medical Center and University of Ulsan College of Medicine, Seoul, South Korea; University of Georgia

**Keywords:** PCR, SARS-CoV-2, bean-extract, gargle, rapid antigen test, saliva

## LETTER

According to Kwon et al. (1), bean extract-based gargle is an effective diagnosis for coronavirus disease 2019 (COVID-19). Although the authors have shown some interesting results, several points are questionable and must be clarified.

First, the methods for Beanguard gargle (BG)-RT-PCR and nasopharyngeal swab (NPS)-RT-PCR are different. Hence, the difference in cycle threshold (Ct) values between BG-RT-PCR and NPS-RT-PCR can be attributed to the difference in the protocols. They used two PCR protocols as follows: Allplex 2019 nCoV real-time PCR (Allplex; Seegene, Seoul, South Korea) that employs three PCR targets (*E*, *RdRp*, and *N*); STANDARD M nCoV real-time PCR (Standard M; SD Biosensors, Suwon, South Korea) that employs only two targets (*E* and *RdRp*). It was not clearly described which reagent was used for which sample. However, in the raw data provided in Supplementary Appendix 1, three Ct values were shown for the NPS-RT-PCR of *E*, *RdRp*, and *N*, while two were shown for the saliva RT-PCR Ct for *E* and *RdRp*. It seems that they used the Allplex assay for nasopharyngeal swabs and the STANDARD M assay for saliva. Both assays employ 40 amplification cycles. However, because the STANDARD M assay includes five preamplification cycles, it results in lower Ct values for the same RNA amount compared with the Allplex assay (2). In the external quality assessment of severe acute respiratory syndrome coronavirus 2 (SARS-CoV-2), the STANDARD M assay shows Ct values 3 to 4 times lower compared to the Allplex assay (3). Therefore, the Ct value difference of 2 can be attributed to the difference between the two assays.

Second, the authors did not use the appropriate controls for the comparison between BG-RT-PCR and NPS-RT-PCR. They reported that BG-RT-PCR shows lower Ct values by about 2 compared to NPS-RT-PCR and the difference was statistically significant. Although a clear conclusion was not drawn from the results, it might be the basis of the claims that bean extract efficiently captured viral nucleic acids in saliva. However, they did not compare bean-extract gargled saliva to control samples, such as gargled saliva alone. Therefore, the effect of the bean-extract gargle on saliva remains unknown.

Third, the number of asymptomatic patients used in the study is too small. The asymptomatic patients make up only 27% of the patients. Moreover, in the evaluation of diagnostic performance, the specimens from asymptomatic patients contribute to only 7% (11/156) of the samples. Saliva is easy to collect and suitable for screening asymptomatic patients, as the authors have described. The retrospective design of this study has limited its potential to help validate the actual performance of the bean extract gargle. A small fraction of specimens from asymptomatic patients made it less reliable.

Every scientific experiment should have controls other than independent variables. In this case, the authors compared the specimens but ignored the difference in the PCR protocols. It is well known that the difference in these two assays can affect Ct values even with the same amount of SARS-CoV-2 RNA.

For the reasons described above, we think that the results of the publication of this research should be interpreted more cautiously.
